# Acute Kidney Disease and Mortality in Acute Kidney Injury Patients with COVID-19

**DOI:** 10.3390/jcm10194599

**Published:** 2021-10-06

**Authors:** Filipe Marques, Joana Gameiro, João Oliveira, José Agapito Fonseca, Inês Duarte, João Bernardo, Carolina Branco, Claúdia Costa, Carolina Carreiro, Sandra Braz, José António Lopes

**Affiliations:** Division of Nephrology and Renal Transplantation, Department of Medicine, Centro Hospitalar Universitário Lisboa Norte, EPE, 1649-028 Lisbon, Portugal; joana.estrelagameiro@gmail.com (J.G.); joaomoliveira@campus.ul.pt (J.O.); jose.nuno.agapito@gmail.com (J.A.F.); ines.cc.duarte@gmail.com (I.D.); joaofbernardo@gmail.com (J.B.); carolinagbranco@hotmail.com (C.B.); claucosta_30@hotmail.com (C.C.); ctc.1991@gmail.com (C.C.); sandrabbraz@gmail.com (S.B.); jalopes93@hotmail.com (J.A.L.)

**Keywords:** acute kidney injury, acute kidney disease, COVID-19

## Abstract

Background: The incidence of AKI in coronavirus disease 2019 (COVID-19) patients is variable and has been associated with worse prognosis. A significant number of patients develop persistent kidney damage defined as Acute Kidney Disease (AKD). There is a lack of evidence on the real impact of AKD on COVID-19 patients. We aim to identify risk factors for the development of AKD and its impact on mortality in COVID-19 patients. Methods: Retrospective analysis of COVID-19 patients with AKI admitted at the Centro Hospitalar Universitário Lisboa Norte between March and August of 2020. The Kidney Disease Improving Global Outcomes (KDIGO) classification was used to define AKI. AKD was defined by presenting at least KDIGO Stage 1 criteria for >7 days after an AKI initiating event. Results: In 339 COVID-19 patients with AKI, 25.7% patients developed AKD (*n* = 87). The mean age was 71.7 ± 17.0 years, baseline SCr was 1.03 ± 0.44 mg/dL, and the majority of patients were classified as KDIGO stage 3 AKI (54.3%). The in-hospital mortality was 18.0% (*n* = 61). Presence of hypertension (*p* = 0.006), CKD (*p* < 0.001), lower hemoglobin (*p* = 0.034) and lower CRP (*p* = 0.004) at the hospital admission and nephrotoxin exposure (*p* < 0.001) were independent risk factors for the development of AKD. Older age (*p* = 0.003), higher serum ferritin at admission (*p* = 0.008) and development of AKD (*p* = 0.029) were independent predictors of in-hospital mortality in COVID-19-AKI patients. Conclusions: AKD was significantly associated with in-hospital mortality in this population of COVID-19-AKI patients. Considering the significant risk of mortality in AKI patients, it is of paramount importance to identify the subset of higher risk patients.

## 1. Background

Coronavirus disease 2019 (COVID-19) pandemic caused by severe acute respiratory syndrome-coronavirus-2 (SARS-CoV-2) was first identified in December 2019 in Wuhan, China and rapidly spread across the globe, being responsible for more than 130 million infections and almost 3 million deaths worldwide until the beginning of April 2021 [1].

Acute kidney injury (AKI) has been reported as a severe complication of COVID-19 with a higher risk of mortality [2]. Although the reported incidence of AKI among hospitalized patients with COVID-19 varies widely, with rates as high as 36–46% [3,4,5]. Although pathophysiology of AKI in COVID-19 has not been fully elucidated, histopathologic examination from autopsied kidney tissue shows SARS-CoV-2 viral particles in podocytes and renal tubular cells, implying a direct infection of the kidney [6,7,8]. Collapsing glomerulopathy has also been described [9,10,11]. In addition, dehydration, rhabdomyolysis, hypoxemia, the presence of underlying disease and improper administration of nephrotoxic substances can further contribute to renal failure in COVID-19 patients [12,13].

AKI in COVID-19 infected patients is associated with poor outcomes, being an independent risk factor for mortality [5,14,15,16]. Latest reports suggest that the prognosis of COVID-19 AKI patients is worst in those with Stage 3 AKI and with persistent kidney damage [17,18,19]. Although AKI and Chronic Kidney Disease (CKD) are well-characterized entities, vastly studied in COVID-19 patients, there are a significant number of patients who developed a persistent kidney damage, with delayed recovery, during the course of the disease, with implications on the prognosis [4,5,20]. Acute Kidney Disease (AKD) has been proposed to define the course of disease after AKI among patients in whom the renal pathophysiologic processes are ongoing, representing a period of time window wherein critical interventions might be initiated to alter the natural history of kidney disease [21,22] and has been associated with higher progression to CKD and mortality [23].

Knowing the risk factors for AKD, we can identify patients at high risk and implement early strategies for preventing or mitigate the impact of COVID-19-associated kidney injury, such as eviction of nephrotoxic substances or optimization of the volemic state.

There is a lack of evidence on the real impact of AKD on COVID-19 patients. In this retrospective analysis, we aimed to identify risk factors for the development of AKD and to analyze the impact of AKD on mortality of COVID-19 patients.

## 2. Materials and Methods

This is a single-center retrospective analysis of COVID-19-AKI patients admitted at the Centro Hospitalar Universitário Lisboa Norte (CHULN) between March and August of 2020. This study was approved by the Ethical Committee in agreement with institutional guidelines. Due to the retrospective and noninterventional nature of the study, informed consent was waived by the Ethical Committee.

### 2.1. Participants

Eligible patients were selected as adult patients (≥18 years of age) who tested positive by polymerase chain reaction (PCR) testing of a nasopharyngeal sample for COVID-19 and were admitted to a Dedicated Unit for COVID-19 patients (UICIVE) and developed AKI during hospital stay. We excluded CKD patients on renal replacement therapy and patients who were discharged or died less than one week after hospital admission.

### 2.2. Variables and Outcomes

Patient variables were collected from individual clinical records.

The following variables were analyzed: patient demographic characteristics (age and gender); comorbidities (hypertension, diabetes mellitus, cardiovascular disease (CVD), chronic kidney disease (CKD), chronic obstructive pulmonary disease (COPD), cirrhosis and/or malignancy); previous treatment with renin-angiotensin-aldosterone system (RAAS) inhibitor; laboratory values at admission (serum hemoglobin, c-reactive protein (CRP), serum albumin, SCr, arterial blood gas, lactate level and pH analysis); development of ARDS and treatment with hydroxychloroquine, lopinavir/ritonavir, tocilizumab, corticosteroids and remdesivir during hospital admission; nephrotoxin exposure, mechanical ventilation, vasopressor use and requirement for renal replacement therapy.

The outcomes measured were the development of AKD and in-hospital mortality.

### 2.3. Definitions

The Kidney Disease Improving Global Outcomes (KDIGO) classification according to both serum creatinine (SCr) and urine output (UO) criteria was used to define AKI [21]. Pre-admission SCr (SCr within the previous three months) was considered as baseline value. When unavailable, baseline SCr was estimated from the MDRD equation [21], accepting the lower limit of a normal baseline GFR of 75 mL/min/1.73 m^2^.

AKD was defined by presenting at least KDIGO Stage 1 criteria for >7 days after an AKI initiating event [22].

For the diagnosis of COVID-19, it was required at least one positive PCR test for SARS-CoV-2, collected either from nasal exudate or bronchial secretions.

Diabetes mellitus was diagnosed according to the American Diabetes Association criteria [24], and hypertension was diagnosed according to the seventh report of the Joint National Committee [25]. CKD was defined according to KDIGO classification [26]. Chronic Obstructive Pulmonary Disease (COPD) comprised emphysema and chronic bronchitis, and Cardiovascular Disease (CVD) was considered as present whenever a history of cerebrovascular disease, chronic heart failure of any cause, cardiac ischemic disease and/or peripheral arterial disease was documented; also, a previous diagnosis on clinical records was considered sufficient for the confirmation of these diagnosis.

RASS inhibitors were angiotensin II receptor blockers and angiotensin conversion enzyme inhibitor. There was need treatment for at least 48 h with hydroxychloroquine, lopinavir/ritonavir, tocilizumab, corticosteroids and remdesivir to be considered as COVID-19 treatment.

Anemia was defined as hemoglobin <12 g/dL in women and <13 g/dL in men. Hypoalbuminemia was defined as serum albumin < 3.5 g/dL and acidemia as a serum pH < 7.35. ARDS was diagnosed according to the 2012 Berlin definition of ARDS [27,28].

### 2.4. Statistical Methods

Categorical variables were described as the total number and percentage for each category, whereas continuous variables were described as the mean ± standard deviation. Normally distributed continuous variables were compared with the Student’s t-test, non-normally distributed continuous variables were compared with the Mann–Whitney U test, and categorical variables were compared with the chi-square test.

We performed univariate analysis in all variables to determine statistically significant factors that may have contributed to the development of AKD and in-hospital mortality. Only variables with a significant statistical difference were included in the multivariate analysis using the Cox logistic regression method.

Data were expressed as Odds Ratios (ORs) with 95% Confidence Intervals (CIs). Statistical significance was defined as a *p*-value <0.05. Statistical analysis was performed with the statistical software package SPSS for Windows (version 21.0).

## 3. Results

Between March and August of 2020, we screened a total of 544 SARS-CoV-2-infected patients admitted at the UICIVE. In this cohort, 62.3% developed AKI during hospital stay (*n* = 339). Patients with AKI were older (71.7 ± 17.0 vs. 64.2 ± 18.4, *p* < 0.001) and were more likely to have previous comorbidities such as arterial hypertension (72.1% vs. 50.0%, *p* < 0.001), diabetes (31.2% vs. 20.0%, *p* = 0.016), cerebrovascular disease (39.1% vs. 21.5%, *p* < 0.001) and chronic kidney disease (26.1% vs. 7.9%, *p* < 0.001).

Demographic variables, clinical and laboratory characteristics are described in Table 1.

In this cohort of COVID-19 patients with AKI, mean age was 71.7 ± 17.0 years, and the majority were male (56.3%). Seventy percent of patients were hypertensive, 30.4% diabetic, 38.0% had CVD, 12.7% had COPD, 25.4% had CKD, and 15.6% had malignancy. In addition, 157 patients (46.3%) were previously medicated with a RAAS inhibitor. During hospital admission, 28.0% of patients received hydroxychloroquine, 36.3% received lopinavir/ritonavir, 28.6% received corticosteroids and 10.3% received remdesivir. Baseline Scr was 1.03 ± 0.44 mg/dL, with a mean eGFR of 71.0 ± 24.6 mL/min/1.73 m^2^. Here, 56 patients (16.5%) had a Brescia Score ≥2. At the hospital admission, mean SCr was 1.60 ± 1.76 mg/dL, hemoglobin was 12.6 ± 2.3, serum albumin was 3.62 ± 0.50 mg/dL, CRP was 10.33 ± 9.77 mg/dL, and lactate level was 14.9 ± 10.2 mg/dL. Anemia was present in 42.5% of patients at admission and acidemia in 35.8%.

The majority of patients were classified as KDIGO stage 3 (54.3%), 13.6% KDIGO had stage 2 and 32.2% had KDIGO stage 1. Of the patients with AKI, 25.7% developed AKD (*n* = 87).

During hospital admission, 87 patients required intensive care unit (ICU) admission (25.7%), 15.0% patients required mechanical ventilation, 4.1% required vasopressors, 11.8% developed ARDS and 27.1% required renal replacement therapy (RRT). Fifty patients (14.7%) were exposed to nephrotoxins. The mean Scr at hospital discharge was 1.12 ± 0.81 mg/dL. Three patients were dependent on RRT at hospital discharge. These patients had a length of stay in hospital of 33.6 ± 44.3 days. The in-hospital mortality in this cohort of COVID-19-AKI patients was 18.0%.

### 3.1. Acute Kidney Disease

Patients who developed AKD had higher incidence of hypertension (82.8% vs. 65.0%, *p* = 0.002) and CVD (44.8% vs. 32.1%, *p* = 0.033). CKD was also more frequent patients who developed AKD (20.3% vs. 13.5%, *p* < 0.001), with higher baseline Scr (1.33 ± 0.66 vs. 0.94 ± 0.27, *p* < 0.001). At hospital admission, patients who developed AKD had more frequently a Brescia Score ≥2 (24.1% vs. 11.8%, *p* = 0.004), hypoalbuminemia (63.2% vs. 42.6%, *p* = 0.003) and acidemia (21.8% vs. 7.6%, *p* < 0.001), lower hemoglobin (12.1 ± 2.5 vs. 12.8 ± 2.2%, *p* = 0.011) and lower CRP (7.90 ± 7.56 vs. 11.1 ± 10.2, *p* = 0.009). Both vasopressor use (9.2% vs. 2.1%, *p* = 0.005) and exposure to nephrotoxins (26.4% vs. 10.1%, *p* < 0.001) were associated with AKD development. Patients who developed AKD were more frequently treated with hydroxychloroquine (43.7% vs. 23.6%, *p* < 0.001) but less frequently with corticosteroids (20.7% vs. 33.3%, *p* = 0.040) and remdesivir (1.1% vs. 14.3%, *p* = 0.001).

Discharge Scr was higher in patients who developed AKD (1.82 ± 1.12 vs. 0.82 ± 0.23, *p* < 0.001). The in-hospital mortality was significantly higher in AKD patients (34.5% vs. 6.8%, *p* < 0.001). (Table 1)

An adjusted multivariate analysis to demographic, clinical, hospital admission variables and during hospital stay factors was conducted, in which hypertension (*p* = 0.006) and CKD (*p* < 0.001) were independent predictors of development of AKD in COVID-19-AKI patients. Lower hemoglobin (*p* = 0.034) and lower CRP (*p* = 0.004) at hospital admission and nephrotoxin exposure (*p* < 0.001) were also independent predictors for the development of AKD in COVID-19-AKI patients in the multivariable analysis (Table 2).

### 3.2. In-Hospital Mortality

The in-hospital mortality in this cohort of COVID-19-AKI patients was 18.0%.

After multivariate analysis, older age (81.6% vs. 69.6%, *p* = 0.010; unadjusted OR 1.06 (95% CI 1.04–1.09), *p* < 0.001; adjusted OR 1.08 (95% CI 1.03–1.14), *p* = 0.003), higher serum ferritin at admission (1738.9 vs. 1137.1, *p* = 0.014; unadjusted OR 1.00 (95% CI 0.99–1.00), *p* = 0.045; adjusted OR 1.00 (95% CI 0.99–1.00), *p* = 0.008), and development of AKD (34.5% vs. 6.8%, *p* < 0.001; unadjusted OR 7.27 (95% CI 3.71–14.25), *p* < 0.001; adjusted OR 3.06 (95% CI 1.12–8.35), *p* = 0.029) were independent predictors of in-hospital mortality in COVID-19-AKI patients (Table 3).

## 4. Discussion

In this retrospective cohort of 339 COVID-19 patients who developed AKI during hospital admission, AKD was an independent predictor of mortality. CKD and hypertension were risk factors for the development of AKD, and there was also a tendency for older age as a risk factor for AKD. CKD and hypertension had already been reported as risk factors for AKI in COVID-19 patients [20,28,29]. These findings enhance the impact of advanced age, CKD and hypertension as major risk factors for acute kidney damage, with higher incidence seen in patients who developed a more severe and persistent disease.

The impact of AKI on long-term renal function decline and mortality has been previously reported [30,31,32,33]. In this study, we demonstrated that AKD patients were a subpopulation of AKI patients with increased risk of in-hospital mortality.

The impact of the renal recovery after AKI on patients’ outcomes has increasingly become the focus of research [34]. Omotoso et al. [35] retrospectively studied a population of 11,538 hospitalized patients with AKI and found that complete renal recovery after an episode of AKI in patients with normal baseline kidney function is associated with a lower risk of long-term major adverse cardiovascular events when compared with those who did not fully recover. Pannu et al. [36] also found that renal recovery after AKI is associated with a lower risk of death or adverse renal outcomes after hospital discharge. The negative impact of delayed renal recovery in AKI on mortality also seems to be appliable to COVID-19 patients.

AKI corresponds to a rapidly decrease in kidney function that occurs over a period ≤7 days, and CKD to an abnormality in kidney function or structure that lasts >90 days. They share the main risk factors, and there is increasing evidence that AKI and CKD likely represent a continuum, with patients who have a sustained episode of AKI having an increased risk for developing or worsening CKD [37].

In 2017, the Acute Disease Quality Initiative 16 Workgroup emphasized the importance of AKD as a distinct entity from AKI and CKD, corresponding to the course of disease after AKI among patients in whom the renal pathophysiologic processes are ongoing [22]. Peeraponaratana et al. [38] showed that AKD was common amongst septic-shock patients and was associated lowest rates of renal function recovery prior to discharge when compared with those of developed AKI without persistently decreased kidney function. Similar to what was seen in our study, CKD patients were more predominant in patients developing AKD. Xiao et al. [39] also demonstrated AKD was independently associated with increased 90-day mortality in hospitalized AKI patients. Gameiro et al. [40] reported that AKD after AKI was independently associated with the risk of long-term need for dialysis and/or renal function decline and with the risk of death after hospital discharge in a population of 256 septic AKI patients admitted to an ICU.

Despite these previous studies, that highlighted the importance of AKD as a risk factor for in-hospital mortality and major renal outcomes, little is known about the impact of AKD in COVID-19 patients. This is the first study that associated AKD as an independent risk factor for mortality in COVID-19 patients. Similar to septic AKD patients, COVID-19 patients that developed AKD could also be a population with higher risk for poor renal outcomes, such as the long-term need for dialysis or renal function decline.

Lower hemoglobin and presence of hypoalbuminemia were associated with the development of AKD in COVID-19-AKI patients, in the univariate analysis, but only hypoalbuminemia persisted as a risk factor in the multivariate analysis. Hypoalbuminemia has already been implicated as risk factor for AKI and progression to CKD [41,42] and an early predictor of mortality and adverse events in COVID-19 infection [43]. It could be a sign of poor nutritional status, which could mean that these patients are more fragile, and so, more prone to develop persistent kidney damage. Anemia as also been implicated as a risk factor for AKI in critically ill patients [44]. Lower hemoglobin level could also suggest a more fragile patient, with greater burden of chronic comorbidities, more prone to delay renal recovery [45]. However, we also know that COVID-19 could cause anemia and that albumin level decreases in severe inflammation [46,47,48], meaning that they can also be markers of a more severe disease, with increased risk of persistent kidney damage. The presence of anemia and hypoalbuminemia did not reach statistical significance in the multivariate analysis probably due to the size of the relatively reduced population analyzed.

In this cohort, higher serum ferritin was independently associated with mortality after multivariate analysis. Several authors already reported the association between higher serum ferritin and mortality [49,50,51]. Many inflammatory biomarkers, such as interleukin (IL)-2, IL-6, IL-10 and tumor necrosis factor, were found higher in COVID-19 patients with severe disease, compared with those with mild or moderate disease [52]. Studies have shown that the exacerbated inflammatory response (cytokine storm) can directly impair organ function in COVID-19 patients with moderate to severe disease, leading to decompensation, organ dysfunction and death [53]. Ferritin also plays a critical role in inflammatory response; therefore, it could represent another important marker of hyperinflammatory state and disease severity, associated with higher mortality.

As expected, the presence of acidemia and SCr at hospital admission was associated with the development of AKD in the univariate analysis; however, they did not reach statistical significance in the multivariate analysis, which could be due to the small population analyzed. Higher incidence of acidemia and SCr at hospital admission could suggest a more severe AKI, and so, higher probability for developing persistent kidney damage, consequently with higher incidence of AKD. The same could be said to the administration of nephrotoxins during hospital stay, which was independently associated with the development of AKD in COVID-19-AKI patients. The administration of nephrotoxins during hospital stay could provoke or worsen an already established acute kidney damage, with implications on the severity and recovery of the kidney injury [54,55].

The administration of remdesivir was associated with lower incidence of AKD in the univariate analysis, but we need to carefully watch this finding. In this center, there was a strict protocol for the use of remdesivir, with a careful selection that excluded patients with more severe comorbidities, and so, only patients with better prognosis would be selected for the administration of this drug, which could explain the substantial difference in the incidence of AKD. Corticosteroids are the only drug class that has showed benefits on the survival of patients with COVID-19 [56]; as such, they are responsible for the improvement on the overall prognosis of these patients, with faster recovery and lesser exposure to biomarkers and drugs that would delay renal recovery. Hydroxychloroquine was frequently used as a second- or third-line therapy, in patients with potentially more severe and prolonged disease, and so, more prone to having severe and continuous kidney damage, which would explain the negative impact of this drug on the development of AKD in AKI-COVID-19 patients.

Previous reports suggested that patients with more severe respiratory disease were at higher risk for developing AKI, with higher incidences seen in patients with ARDS and need for mechanical ventilation [28,57,58,59]. Although patients with AKD had more frequently a Brescia Score ≥2 in this study, suggesting a more severe disease, it was not associated with higher ICU admission, ARDS incidence or need for mechanical ventilation, meaning that the severity of the lung damage did not correlate with the persistence of the kidney damage. On the other hand, the history of CVD and the use of vasopressors, well-known risk factors for AKI [20,60,61,62,63], seem to have a greater impact on the perpetuation of the kidney injury, suggesting a stronger association with acute or chronic CVD and acute kidney damage.

Certain limitations have to be noted. First, the single-center and retrospective nature of the study could compromise the generalization of our results. Secondly, we did not take under consideration patients’ race, which can mask certain ethnical characteristics that could alter kidney recovery. Third, the development of proteinuria and CVD during follow-up was not accounted for, and we know that they are both factors that influence renal function and long-term survival. Fourth, the use of SCr to estimate AKD may overestimate renal recovery in COVID-19 patients due to the loss of muscle mass seen in these patients with prolonged in-hospital stay. Fifth, we did not analyze causes of mortality. Finally, due to the short time of follow-up, we did not evaluate long-term patient and kidney outcomes, which could underestimate the true impact of AKD on this population.

Despite these limitations, our study has several notable strengths. To the best of our knowledge, this is the first study evaluating the association between AKD, as defined by the ADQI 16 work group, and in-hospital mortality in COVID-19-AKI. It also the first study that addresses risk factors for the development of AKD in COVID-19 AKI patients. The study had a significant number of participants in a selected population, empowering the conclusions in this group of COVID-19-AKI patients. Finally, most studied variables were routinely registered during daily clinical practice.

## 5. Conclusions

To conclude, we identified AKD as an independent predictor of in-hospital mortality in COVID-19-AKI patients. Furthermore, we identified risk factors for AKD development, namely prior diagnosis of hypertension and CKD, lower hemoglobin and lower CRP at the hospital admission and nephrotoxin exposure during admission.

Considering the significant risk of mortality in AKI patients, it is of paramount importance to identify the subset of higher risk patients. Further investigation of AKD in COVID-19 patients is warranted to identify at-risk populations, in order to employ preventive strategies, guarantee an early diagnosis and prompt adequate treatment, ultimately, to improve patients’ prognosis.

## Figures and Tables

**Table 1:** Patients’ baseline characteristics and according to AKD development.

Characteristic	Baseline (*n* = 339)	No-AKD (*n* = 237)	AKD (*n* = 87)	*p*-Value
Age (year)	71.7 ± 17.0	70.0 ± 17.3	74.1 ± 17.0	0.055
Gender (Male)—*n* (%)	191 (56.3)	132 (55.7)	53 (60.9)	0.400
Comorbidities—*n* (%)				
Hypertension	238 (70.2)	154 (65.0)	72 (82.8)	0.002
Diabetes	103 (30.4)	70 (29.5)	28 (32.2)	0.646
CVD	129 (38.0)	76 (32.1)	39 (44.8)	0.033
CKD	86 (25.4)	32 (13.5)	48 (20.3)	<0.001
COPD	43 (12.7)	32 (13.5)	8 (9.2)	0.296
Cirrhosis	14 (4.1)	13 (5.5)	2 (2.3)	0.455
Neoplasia	53 (15.6)	31 (13.1)	15 (17.2)	0.342
RAAS inhibitors—*n* (%)	157 (46.3)	108 (45.6)	43 (49.4)	0.541
Baseline SCr (mg/dL)	1.03 ± 0.44	0.94 ± 0.27	1.33 ± 0.66	<0.001
Baseline eGFR (mL/min/1.73 m^2^)	71.0 ± 24.6	76.1 ± 22.4	57.5 ± 24.7	<0.001
Brescia Score ≥ 2	56 (16.5)	28 (11.8)	21 (24.1)	0.004
Laboratory				
Admission SCr (mg/dL)	1.60 ± 1.76	1.29 ± 0.76	2.31 ± 2.68	<0.0011
Hemoglobin (g/dL)	12.6 ± 2.3	12.8 ± 2.2	12.1 ± 2.5	0.011
Anemia—*n* (%)	144 (42.5)	92 (38.8)	44 (50.6)	0.057
Serum albumin (g/dL)	3.62 ± 0.50	3.50 ± 0.51	3.36 ± 0.61	0.072
Hypoalbuminemia—*n* (%)	162 (47.8)	101 (42.6)	55 (63.2)	0.003
Serum ferritin (ug/dL)	1255.9 ± 1547.5	1243.8 ± 1305.2	1228.0 ± 1835.5	0.947
CRP (mg/dL)	10.33 ± 9.77	11.1 ± 10.2	7.90 ± 7.56	0.009
Acidemia—*n* (%)	38 (11.2)	18 (7.6)	19 (21.8)	<0.001
Lactate level (mg/dL)	14.9 ± 10.2	14.2 ± 7.4	14.3 ± 8.7	0.972
Nephrotoxins—*n* (%)	50 (14.7)	24 (10.1)	23 (26.4)	<0.001
ICU admission—*n* (%)	87 (25.7)	64 (27.0)	21 (24.1)	0.589
Mechanical ventilation—*n* (%)	51 (15.0)	33 (13.9)	16 (18.4)	0.353
Vasopressor use—*n* (%)	14 (4.1)	5 (2.1)	8 (9.2)	0.005
ARDS—*n* (%)	40 (11.8)	26 (11.0)	12 (13.8)	0.515
COVID-19 treatment				
Hydroxychloroquine—*n* (%)	95 (28.0)	56 (23.6)	38 (43.7)	<0.001
Lopinavir/ritonavir—*n* (%)	123 (36.3)	83 (35.0)	40 (46.0)	0.076
Tocilizumab—*n* (%)	12 (3.5)	11 (4.6)	1 (1.1)	0.143
Corticosteroids—*n* (%)	97 (28.6)	79 (33.3)	18 (20.7)	0.040
Remdesivir—*n* (%)	35 (10.3)	34 (14.3)	1 (1.1)	0.001
Persistent AKI—*n* (%)	186 (54.9)	84 (35.4)	47 (54.0)	0.005
KDIGO stage 1—*n* (%)	109 (32.2)	60 (25.3)	23 (26.4)	
KDIGO stage 2—*n* (%)	46 (13.6)	18 (7.6)	13 (14.9)	0.183
KDIGO stage 3—*n* (%)	184 (54.3)	105 (44.3)	36 (41.4)	
RRT requirement—*n* (%)	53 (15.6)	27 (11.4)	14 (16.1)	0.338
Mortality within the first week—*n* (%)	15 (4.4)	-	-	-
AKD—*n* (%)	87 (25.7)	-	-	-
Discharge SCr (mg/dL)	1.12 ± 0.81	0.82 ± 0.23	1.82 ± 1.12	<0.001
Discharge on HD—*n* (%)	3 (0.9)	1 (0.4)	2 (2.3)	0.096
LOS in hospital (days)	33.6 ± 44.3	34.4 ± 43.3	38.4 ± 49.6	0.475
In-hospital mortality—*n* (%)	61 (18.0)	16 (6.8)	30 (34.5)	<0.001

AKI, acute kidney injury; ARDS, acute respiratory distress syndrome; CRP, c-reactive protein; CKD, chronic kidney disease; CVD, cardiovascular disease; COPD, chronic obstructive pulmonary disease; ICU, intensive care unit; GFR, glomerular filtration rate; LOS, length of stay; NL, neutrophil and lymphocyte; RRT, renal replacement therapy; RAAS, renin angiotensin aldosterone system; SCR, serum creatinine; SOFA, sequential organ failure assessment.

**Table 2:** Univariate and multivariate analysis of factors predictive of AKD in COVID-19 patients.

Characteristic	AKD			
	Unadjusted OR (95% CI)	*p*-Value	Adjusted OR (95% CI)	*p*-Value
Age	1.01 (1.00–1.03)	0.073		
Gender (Male)	1.26 (0.76–2.08)	0.364		
Comorbidities				
Hypertension	2.59 (1.40–4.80)	0.003	2.95 (1.36–6.38)	0.006
Diabetes	1.16 (0.68–1.96)	0.593		
CVD	1.66 (1.00–2.74)	0.049		
CKD	7.57 (4.32–13.26)	<0.001	6.17 (2.56–19.43)	<0.001
COPD	0.65 (0.29–1.47)	0.299		
Cirrhosis	0.43 (0.10–1.91)	0.269		
Neoplasia	1.38 (0.71–2.71)	0.343		
RAAS inhibitors	1.17 (0.71–1.91)	0.541		
Lab at admission				
Serum Cr	1.96 (1.46–2.63)	<0.001	1.25 (0.97–1.61)	0.087
Hemoglobin	0.87 (0.78–0.97)	0.010	0.87 (0.76–0.99)	0.034
NL ratio	1.00 (0.97–1.04)	0.903		
Serum albumin	0.65 (0.39–1.06)	0.086		
Serum ferritin	1.00 (1.00–1.00)	0.972		
CRP	0.96 (0.93–0.99)	0.010	0.95 (0.91–0.98)	0.004
Acidemia	3.37 (1.67–6.80)	0.001	1.45 (0.61–3.67)	0.375
Lactate level	1.00 (0.97–1.04)	0.869		
Nephrotoxins	3.24 (1.71–6.13)	<0.001	6.76 (2.82–16.17)	<0.001

AKI, acute kidney injury; ARDS, acute respiratory distress syndrome; CRP, c-reactive protein; CKD, chronic kidney disease; CVD, cardiovascular disease; COPD, chronic obstructive pulmonary disease; ICU, intensive care unit; GFR, glomerular filtration rate; Lab, laboratory; NL, neutrophil and lymphocyte; RRT, renal replacement therapy; RAAS, renin angiotensin aldosterone system; SCR, serum creatinine; SOFA, sequential organ failure assessment.

**Table 3:** Univariate and multivariate analysis of factors predictive of mortality in COVID-19 patients.

Characteristic	Mortality			
	Unadjusted OR (95% CI)	*p*-Value	Adjusted OR (95% CI)	*p*-Value
Age	1.06 (1.04–1.09)	<0.001	1.08 (1.03–1.14)	0.003
Lab at admission				
Serum Cr	1.16 (1.02–1.33)	0.029	1.20 (0.96–1.51)	0.111
Hemoglobin	0.80 (0.70–0.90)	<0.001	0.90 (0.72–1.14)	0.386
Serum albumin	0.41 (0.22–0.75)	0.004	1.02 (0.36–2.91)	0.973
Serum ferritin	1.00 (1.00–1.00)	0.045	1.00 (0.99–1.00)	0.008
CRP	1.00 (1.00–1.03)	0.849		
Acidemia	1.66 (0.76–3.64)	0.205		
Lactate level	1.05 (1.02–1.09)	0.002	1.05 (0.99–1.10)	0.103
Nephrotoxins	1.58 (0.77–3.24)	0.213		
AKD	7.27 (3.71–14.25)	<0.001	3.19 (1.18–8.58)	0.022

AKI, acute kidney injury; ARDS, acute respiratory distress syndrome; CRP, c-reactive protein; CKD, chronic kidney disease; CVD, cardiovascular disease; COPD, chronic obstructive pulmonary disease; ICU, intensive care unit; GFR, glomerular filtration rate; Lab, laboratory; NL, neutrophil and lymphocyte; RRT, renal replacement therapy; RAAS, renin angiotensin aldosterone system; SCR, serum creatinine; SOFA, sequential organ failure assessment.

## Data Availability

Please contact author for data requests.
